# Development and validation of a duplex real-time PCR for the rapid detection and quantitation of HTLV-1

**DOI:** 10.1186/s12985-023-01970-y

**Published:** 2023-01-17

**Authors:** Huimin Ji, Le Chang, Ying Yan, Lunan Wang

**Affiliations:** 1 National Center for Clinical Laboratories, Institute of Geriatric Medicine, Beijing Hospital, National Center of Gerontology, Chinese Academy of Medical Sciences, Beijing, People’s Republic of China; 2 Beijing Engineering Research Center of Laboratory Medicine, Beijing Hospital, Beijing, People’s Republic of China; 3 Graduate School, Peking Union Medical College, Chinese Academy of Medical Sciences, Beijing, People’s Republic of China

**Keywords:** Human T-lymphotropic virus-1, Real-time quantitative PCR, Proviral load value

## Abstract

**Background:**

The HTLV-1 prevalence in China varies geographically, while HTLV-2 infection has rarely been found so far. Proviral load is one of the determining factors of pathogenesis and progression of HTLV-1 related diseases. However, neither molecular assays nor commercial kits are available for HTLV-1 diagnosis in China. The objective of the present study was to develop and validate a TaqMan qPCR assay for HTLV-1 proviral load quantification.

**Results:**

A plasmid containing both the HTLV-1 of interest and a fragment of the RNase P (RPPH1) gene was constructed and used to establish the standard curves. The assay has a wide dynamic range (2.5 × 10^8^ copies/reaction ~ 25 copies/reaction) and sensitive to 1 copy for HTLV-1 and RPPH1. The limit of detection for Hut102 cell concentration was 0.0218% (95% confidence interval 0.0179–0.0298%). The assay gave coefficient of variation (CV) for both the HTLV-1 and RPPH1 Ct values. All of the HTLV-1 sero-negative samples and MOT cell line (infected with HTLV-2) amplified only the RPPH1 gene by our method, presenting 100% specificity. 85 Samples confirmed positive or indeterminate by LIA were performed by established qPCR assay and WB. 90.0% (27/30) of LIA-HTLV-1-positive, 33% (2/6) of LIA-untypeable and 2% (1/49) of LIA-indeterminate samples were defined as qPCR-positive. The median PVL of LIA-positive samples (n = 27, 1.780 copies/100 cells) was much higher than that of LIA-untypeable and (n = 2, 0.271 copies/100 cells) indeterminate samples (n = 1, 0.017 copies/ 100 cells). Additionally, compared to WB, the duplex qPCR verified more positive samples, demonstrating a better sensitivity.

**Conclusion:**

The duplex qPCR developed here with high sensitivity, good specificity and reproducibility could accurately and quantitatively detect the HTLV-1 PVLs, which can be used to confirm the initial reactive samples for an improved cost/benefit ratio as well as to monitor the clinical progression and efficacy of therapy in patients with HTLV-1 related disease.

**Supplementary Information:**

The online version contains supplementary material available at 10.1186/s12985-023-01970-y.

## Introduction

HTLV-1 is the first RNA retrovirus found to be mainly transmitted by mother-to-child, sexual contact and injecting drug users [1, 2]. It also can be transmitted by the transfusion of infected blood components, liver, kidney and lung transplantation [3]. The two most prevalent diseases derived from HTLV-1 are adult T-cell leukemia (ATL) and HTLV-1-associated myelopathy/tropical spastic paraparesis (HAM/TSP), but HTLV-2 is infrequently linked to a disease [4, 5]. At present, no vaccine is yet available and poor prognosis has been reported for ATL and HAM/TSP. In this sense, screening blood and organ donors has been shown to be a successful tactic for halting the spread of HTLV-1.

According to the report of a statewide multicenter large-scale survey on the seroprevalence of HTLV infection among Chinese blood donors, the majority of mainland China is classified as an HTLV non-endemic area [6]. The National Health Commission of the People's Republic of China has recommended that beginning in 2022, ELISA testing for the presence of the HTLV-1/2 antibody be done on 100% of blood donors in areas with a relatively high prevalence of HTLV (higher than 0.028‰) and on 10–30% of blood donors in areas with a moderate prevalence of HTLV (higher than 0.01‰). And then the blood centers would confirm any initially reactive samples. This program will lessen the burden, lower the cost of screening, and minimize the hazards of HTLV-1 transmission through blood transfusion. For some other countries, especially those with a high need for testing or a shortage of laboratory supplies, pooling of samples for seroepidemiological surveillance of HTLV-1/2 is another strategy to reduce the cost and technician time. However, there will inevitably occur numbers of false positives in the initial screening for the non-endemic regions [7]. Western blot (WB) and line immunoassay (LIA) with high specificity are two of the most frequently used confirmatory assays for the confirmation and differentiation of HTLV-1 and HTLV-2 infections. Whereas, the serologic confirmatory tests are too expensive to be employed in every blood center. In addition, both of them were able to produce a considerable high proportion of inconclusive (indeterminate and untypeable) results, especially when samples obtained from patients with HTLV-2 infection and those from patients coinfected with HIV, and/or HBV, and/or HCV [8]. Molecular assays, such as real-time quantitative PCR (qPCR), with high sensitivity and low cost should be performed to detect the nucleic acids of HTLV-1/2 provirus for confirmation. Since HTLV-1 infects only through cell-to-cell transmission, most previous studies used peripheral blood mononuclear cells (PBMC) for HTLV-1 quantification, and few reports used whole blood [9–12]. Unfortunately, there are presently no commercial kits or sensitive method available to determinate the proviral load (PVL) and assess the real status of HTLV-1 infections in China. More importantly, it has been revealed that more than 4 copies/100 PBMCs is an independent risk factor for progression of ATL, and other investigations have confirmed the significance of quantifying PVL for the assessment of the emergence of HTLV-1-associated diseases [13, 14]. To address the shortcomings of the present confirmative test and offer accurate PVL for better monitoring the affected patients, it is important to create a nucleic acid test with high sensitivity, specificity, and reproducibility.

PVL quantification was initially performed using semiquantitative PCR and southern blots, however neither method was able to determine the precise copy number. In recent years, qPCR has been performed for HTLV-1 or/and HTLV-2 viral quantification. However, the published qPCR techniques had a few shortcomings. (1) the preparation of quantitative standards, which typically involved sequential reference DNA diluents derived from HTLV-1-infected cell lines, was a labor-intensive technique that made it challenging to replicate the findings [9, 10]. (2) the standard curves were typically created using a combination of two distinct plasmids. However, it was challenging to precisely dilute two plasmids, which led to inconsistent findings between laboratories [11]. (3) Prior validation for the majority of the established technologies was insufficient, which was required before the technique could be used in ordinary laboratory settings [11]. (4) the performance of the established methods has rarely been assessed in blood donors, especially in non-endemic areas. (5) the majority of the primer–probe set used for internal reference in earlier approaches came from commercial kits, or the reference gene needed to count cells had to be found independently, which significantly reduced the cost-effectiveness of HTLV-1 PVL quantification [9, 11].

Due to this, we decided to develop a single-tube qPCR assay to quantify HTLV-1 PVLs in PBMCs, which was normalized by means of the quantitation of a cellular gene RNase P (RPPH1). Multiple pairs of primers and probes were designed and screened based on the HTLV-1 conserved region (*pol* region and *tax* region) and the internal reference RPPH1 gene. A plasmid carrying both the HTLV-1 of interest and a fragment of the RPPH1 gene was used to establish the standard curves. The sensitivity, specificity, reproducibility and accuracy of this assay were analyzed and preliminary results on its use for specimens with LIA-indeterminate and positive results from blood donors were reported.

## Methods and methods

### Cells and gDNA preparation

Jurkat, Hut102 and MOT cells were obtained from Peking Union Medical College (PUMC, Beijing, China). Hut102 is a HTLV-1-infected cell line and MOT is a HTLV-2-infected cell line. All of these cells were cultured in RPMI-1640 medium (Gibco), without addition of antibiotics and extra growth factors but supplemented with 10% FBS (Gibco) in a humidified atmosphere with 5% CO2. gDNA was extracted using a Tiangen Magnetic Blood Genomic DNA Kit (Tiangen Biotech, China).

### PcDNA3.1(-)-HTLV-1-RPPH1 plasmid construction and standard curves preparation

**Fig. 1:**
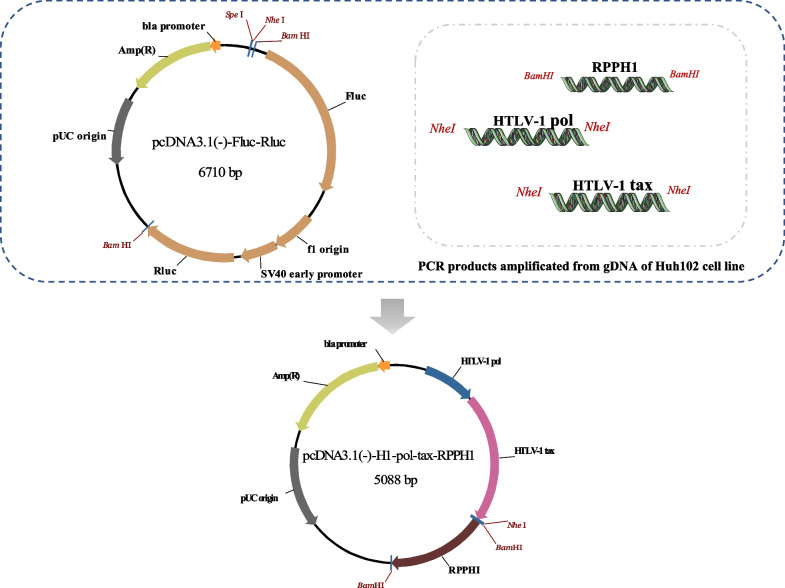
Design of pcDNA3.1(-)-H1-*pol-tax*-RPPH1 plasmid used for quantitation of HTLV-1 proviral load

To construct the pcDNA3.1(-)-HTLV-1-RPPH1 plasmid, the human RPPH1 gene and the HTLV-1 *pol* and *tax* region were amplified using gDNA from Hut102 cells and consecutively cloned into pcDNA3.1(-)-Rluc-Fluc vector (conserved in our lab). The HTLV-1 *pol* and *tax* region were separately cut with SpeI endonucleases and cloned into the vector that contains RPPH1 gene (pcDNA3.1(-)-RPPH1) previously generated by replace with the Fluc and Rluc gene cutting with BamHI endonucleases (Fig. 1). The construct was finally verified by direct DNA sequencing (Shenggong, Shanghai, China). The concentration of the standard plasmid was determined by spectrometry at 260 nm. The pcDNA3.1(-)-H1-*pol*-*tax*-RPPH1 vector was serially diluted at a range of 5 × 10^9^–1 copies/μL in order to establish a quantitative curve. The standard dilutions that from 5 × 10^9^ copies/μL ~ 5 × 10^2^ copies/μL were prepared once and stored at − 20 °C. The final dilutions (50 copies/μL, 5 copies/μL and 0.2 copies/μL) were prepared immediately before use. All the dilutions and individual samples were run in duplicate.

### Selection for specific primers and probes

Primers and probes were designed by Primer 5 to target the conserved sequences of the *pol* and *tax* regions of the HTLV-1 and RPPH1 sequences. Primers were screened by efficiency of amplification using SYBR Premix Ex Taq™ II kit (Takara, Dalian, China), and primers with significant dimerization were removed. Probes were further screened using Hyperstart Premix-UNG (Biori, Zhuhai, China) in accordance with the manufacturer’s instructions. Probes with lower count (Ct) values were chosen for further experiments (data not shown). pcDNA3.1(-)-H1-*pol*-*tax*-RPPH1 was used as template for primers and probes screening. The primers and probes we finally selected for using were listed in Table 1.

**Table 1:** Primers and probes used in the duplex qPCR

Gene	Primer/probe	5'–3' sequences	Amplicon size (bp)
*pol*	HTLV-1-F	CTAGCCCACTTGCAAACTATAGACCT	109
	HTLV-1-R	CGTAGTTACACTGCTGTGGGACAG	
	HTLV-1-P	AGACGCCTTTTTCCAAATCCCCTTACC	
*RPPH1*	RPPH1- F	CATGCCGTCCCGCGATATTGA	102
	RPPH1- R	GTTGATGACGTCAGCGTTCGAATTC	
	RPPH1-P	CTCCGAACCTCTCGCCCTGCC	

### TaqMan real-time quantitative PCR

Real-time qPCRs were conducted using a 7500 ABI (Applied Biosystems). The final concentrations of primers of HTLV-1 *pol* and RPPH1 were optimized from 0.2 to 1.0 μΜ, while the probes were optimized from 0.2 to 1.0 μM. The annealing temperature for qPCR were optimized from 60 to 68 °C. The operate concentration of primers and probes, as well as the annealing temperature selected for the following experiments were based on the high efficiency (between 95 and 105%) and the lowest Ct values at the same concentration of the template. Finally, the optimized amplification was performed using the following cycling conditions: initial step of 2 min/50 °C, denaturation of 5 min/95 °C, 40 cycles of 20 s/95 °C, annealing step of 30 s/60 °C and elongation step of 20 s/72 °C. The final volume of the reaction was 25 μL, the optimized primer concentrations for HTLV-1*pol* and RPPH1 genes were 800 nM, and the optimized probe concentrations for the two genes were 300 nM. The probes were labeled by FAM (HTLV-1 *pol*)/VIC (RPPH1). All reactions were carried out using Hyperstart Premix-UNG (Biori, Zhuhai, China) in a concentration specified by the manufacturer. Standard curves for HTLV-1 and RPPH1 were accepted when the slopes were between − 3.44 and − 3.21 (corresponding to PCR efficiencies of between 95 and 105%) and the coefficients of correlation, r^2^, were > 0.98.

### Performance verification for the established assay

To evaluate the dynamic range of the HTLV-1 *pol* and RPPH1, pcDNA3.1(-)-H1-*pol*-*tax*-RPPH1 plasmid was serially diluted at the concentrations from 5 × 10^9^ to 0.2 copies/μL and each of them was tested in replicate.

To evaluate the intra- and inter-assay reproducibility, our method quantified three plasmid dilutions (10^7^ copies/μL, 10^3^ copies/μL, 5 copies/μL) 6 times per day for 3 consecutive days. Three different cell concentrations of Hut102 with known proviral loads (0.026 copies/100 cells, 3.025 copies/100 cells, 114.7 copies/100 cells) were prepared and each of them were extracted independently for six times. Their gDNA were carried out in the same TaqMan assay to evaluate the accuracy.

To analyze the specificity of the assay, gDNA extracted from HTLV-1-negative specimens (3 were infected with HBV, 3 were infected with HCV, 3 were infected with treponema pallidum, 3 were infected with HIV and 12 were healthy person) and the MOT cell line (infected with HTLV-2) were tested by the assay. Hut102 containing HTLV-1 genome was used as a positive control.

To evaluate the limit of detection (LOD), genomic DNA extracted from a serial dilution of Hut102 cells with Jurkat cells was used as the template, each of which was tested by the duplex qPCR for 20 times. Probit analysis was used to determine the LOD of the assay.

### Digital PCR

For QuantiStudio 3D digital PCR analysis, 5 ng of genomic DNA from Hut102 cells and 10 ng of pcDNA3.1(-)-H1-*pol*-*tax*-RPPH1 plasmid were digested with KpnI (New England Biolabs, Ipswich, MA) was added to digital PCR master mix v2 (Thermo Fisher Scientific, Waltham, MA) with a final concentration of 500 nM forward and reverse primers, and 150 nM of FAM and VIC-labeled probes. The PCR mixture was loaded on the digital PCR chip, and PCR was performed with a LifePro thermal cycler (Bio-Rad) using primers and probes for HTLV-1 and RPPH1 described above and detected with a QX200 droplet reader (Bio-Rad).

### Clinical samples

A total of 85 samples from blood donors were included in this study. All of these samples were confirmed as LIA-positive or indeterminate by INNO-LIA HTLV I/II score kit (including 30 HTLV-1-positive, 6 untypeable and 49 indeterminate samples) and simultaneously tested by WB (MP HTLV Blot 2.4, MP Biomedicals, Singapore) and qPCR. DNA was extracted from the whole blood of each blood sample described as above and HTLV-1 proviral DNA was detected and quantified. The normalized HTLV-1 PVL was calculated as the ratio of (HTLV-1 DNA average copy number/RPPH1 average copy number) × 2 × 100 leukocytes, expressed as the number of HTLV-1 copies per 100 cells.

## Results

### Standard curve and dynamic range of the assay

**Fig. 2:**
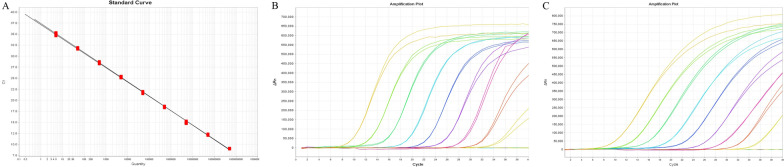
Standard curves for the quantitation of HTLV-1 and RPPH1 gene by qPCR. **A** Amplification plot shows the Log (ΔRn) graphed by the standard curve for HTLV-1 and RPPH1 versus cycle of the qPCR. **B** and **C** PCR characteristics of the standard curve for HTLV-1 and RPPH1

A double-insert plasmid pcDNA3.1(-)-H1-*pol*-*tax*-RPPH1 that contained one copy of the RPPH1 gene and HTLV-1 target region was constructed. To verify the capacity of the plasmid as a standard for quantification of HTLV-1 proviral DNA, serial dilutions from 5 × 10^9^ copies/μL–0.2 copies/μL were tested by the duplex qPCR. Both HTLV-1 *pol* and RPPH1 showed excellent amplification result (slope = 3.252/3.296, Y-intercept = 37.151/37.424, correlation coefficient R^2^ = 0.999/0.999, and efficiency = 103.003/101.102 respectively) when the plasmid dilutions from 5 × 10^7^ copies/μL to 5 copies/μL (Fig. 2). In addition, each set of primer-probes was capable of detectin 1 copy per reaction. As 5 μL template were amplificated by each reaction, we determined the dynamic range of standard curve for both of HTLV-1 and RPPH1 as 2.5 × 10^8^ copies/reaction ~ 25 copies/reaction. As show in Fig. 2, the efficiencies of the HTLV-1 and RPPH1 amplification reactions were similar and the Ct values obtained were identical or almost identical for the same plasmid dilutions. As a result, the plasmid constructed in this study is capable to be as a standard for quantification of HTLV-1 proviral DNA and the RPPH1 gene can be used as an efficient internal standard to normalize the results of the HTLV-1 quantitation.

### Specificity and sensitivity of the assay

24 HTLV-negative samples and the MOT cells with HTLV-2 genome were not amplified for the HTLV-1 *pol* gene but presented amplification of the reference gene RPPH1. Hut102 was amplified for both HTLV-1 *pol* and RPPH1 gene. Therefore, the duplex qPCR assay showed 100% specificity.

In order to quantify the exact copy number of HTLV-1 in per Hut102 cell, both pcDNA3.1(-)-H1-*pol*-*tax*-RPPH1 and Hut102 were subjected to ddPCR. As shown in Table 2, the copy number ratio of the *pol* gene to RPPH1 for pcDNA3.1(-)-H1-*pol*-*tax*-RPPH1 was 1.001, indicating that the ddPCR results were credible and accurate. The mean ratio of the *pol* gene to RPPH1 for gDNA of Hut102 cells was 0.5735, that means each Hut102 cell contains 1.146 copies of HTLV-1. Consequently, gDNAs extracted from a serial dilution of Hut102 cells with Jurkat cells were subjected to our method and each gDNA was tested for 20 replicates. According to the results of probit regression, the limit of detection (LOD) by using our method for Hut102 cell concentration was 0.0218% (95% confidence interval 0.0179–0.0298%), which means the LOD for the HTLV-1 *pol* region was estimated to be 2.5 copies per 10,000 host leukocytes with a 95% hit-rate (Table 3).

**Table 2:** Absolute gene copy number per reaction determined by digital PCR

Sample	Gene copy no./rnx			
	HTLV-1 gene	RPPH1 gene	Ratio of HTLV/RPPH1	Copy no./cell
pcDNA-*pol-tax*-RPPH1	79,800	77,800	1.001	/
100% Hut102	14,340	25,020	0.5735	1.146
4% Hut102/Jurkat	569	37,640	0.0151	0.03025
0.032% Hut102/Jurkat	5.2	39,240	0.00013	0.00026
Jurkat	0	46,720	0	0

**Table 3:** The LoD of cell standards of dual fluorescent qPCR system

Cell concentrations (Hut102/Jurkat) (%)	PVL (copies/100 cells)	Positive results/total tests	Positive rate (%)
20	22.92	20	100
4	4.584	20	100
0.8	0.9186	20	100
0.16	0.183	20	100
0.032	3.67 × 10–2	20	100
0.016	1.83 × 10–2	15/20	75
0.0064	7.33 × 10–3	5/20	25
0.0032	3.67 × 10–3	1/20	5
0	0	0	0

### Intra- and inter-assay reproducibility and accuracy

The reproducibility of the assay was evaluated using three standard dilutions of pcDNA3.1(-)-H1-*pol*-*tax*-RPPH1 plasmid (10^7^ copies/μL, 10^3^ copies/μL, 5 copies/μL). The intra- and inter-assay variations (CVs) for HTLV-1 Ct values were 0.36–2.81% and 0.64–3.19%, respectively. And for RPPH1 were 0.27–0.84% and 0.83–2.55%, respectively (Table 4).

**Table 4:** Intra- and Inter-assay reproducibility of duplex qPCR for detecting HTLV-1 and RPPH1

No. of copies	HTLV-1				RPPH1			
	Intra-assay Ct values		Inter-assay Ct values		Intra-assay Ct values		Inter-assay Ct values	
	Mean SD	CV (%)	Mean SD	CV (%)	Mean SD	CV (%)	Mean SD	CV (%)
10^7^copies/μL	11.70 ± 0.22	1.88	11.74 ± 0.35	3.06	11.92 ± 0.06	0.87	12.01 ± 0.10	0.48
10^3^copies/μL	25.14 ± 0.08	0.3	25.18 ± 0.10	0.39	25.57 ± 0.18	1.31	25.51 ± 0.00	1.55
5 copies/μL	34.97 ± 0.32	0.92	34.99 ± 0.33	0.93	37.04 ± 0.35	1.95	36.94 ± 0.72	2.9

To evaluated the accuracy of the duplex qPCR, three standard Hut102 dilutions with accurate HTLV-1 proviral loads (quantitated by ddPCR) were tested by our method. Each sample’s gDNA was extracted independently by six times and then were carried out in the same TaqMan assay. The CV values of the standard cells were ranged from 2.9% ~ 33.45%, and for Hut102 cells, the detected copy number (1.22 ± 0.035 copies/cell) was very close to the theoretical values (Table 5). These data indicated that the duplex qPCR was accurate and practical for quantifying cellular genes with dual plasmid as a standard.

**Table 5:** the accuracy of the duplex qPCR for detecting HTLV-1 PVLs

HTLV-1 copy no./100 cells detected by ddPCR	HTLV-1 copy no./100 cells detected by duplex qPCR	
	Mean ± SD	CV(%)
0.026	0.0275 ± 0.0092	33.45
3.025	3.4148 ± 0.1833	17.08
114.7	122.1513 ± 3.5478	2.9

**Table 6:** Results of qPCR and WB among LIA-positive and indeterminate samples

LIA	qPCR			WB			
	HTLV-1	Neg	Rate	HTLV-1	Ind	Neg	Rate
HTLV-1	27	3	0.90	27	2	1	0.90
HTLV	2	4	0.33	1	1	4	0.17
Ind	1	48	0.02	0	3	46	0.00

WB, Western blot; LIA, line immunoassay; Ind, indeterminate, Pos, Positive; Neg, Negative

### HTLV-1 proviral load in clinic samples

85 Samples confirmed positive or indeterminate by LIA were tested by the established quantitative assay and WB. Among the 30 LIA-HTLV-1-positive samples, 27 were quantified as qPCR-positive. The PVL of HTLV-1 ranged from 0.001 to 11.397 copies/100 cells. As shown in Table 6, 2 of 27 qPCR-positive samples were confirmed as WB-indeterminate. Otherwise, for the 3 LIA-positive but qPCR-negative samples, one was WB-HTLV-1-positive, one was WB-indeterminate and the other one was WB-negative. It is noteworthy that 2 of 6 LIA-untypeable samples was confirmed as HTLV-1 positive by qPCR. While for WB, one was confirmed as HTLV-1-positive, 1 were indeterminate and the other 4 were negative. In addition, one of the 49 LIA-indeterminate samples was qPCR-positive but WB-negative. The other 48 LIA-indeterminate samples were confirmed as WB-negative (46 samples) or indeterminate (3 samples).

**Fig. 3:**
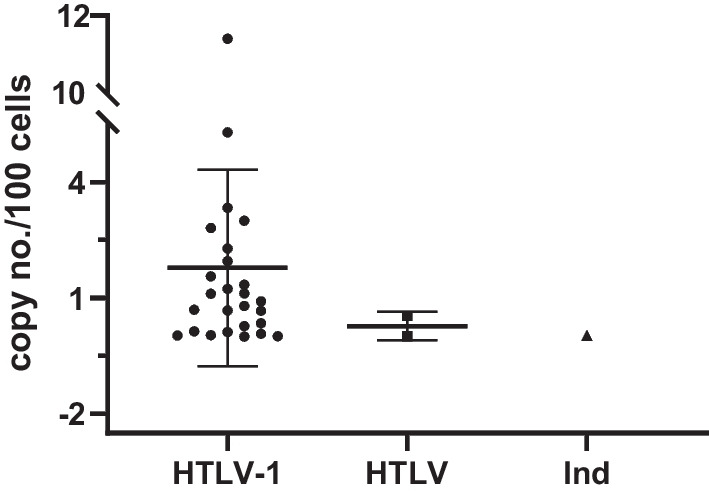
HTLV-1 PVLs determined in HTLV-1 infected blood donors. The median copy number of LIA-positive samples (n = 27, 1.780 copies/100 cells) was higher than that of the untypeable (n = 2, 0.271 copies/100 cells) and indeterminate samples (n = 1, 0.017 copies/100 cells), but the sample sizes of the latter two were too small to be statistically analyzed

Of all the qPCR-positive samples, the median copy number of LIA-positive samples (n = 27, 1.780 copies/100 cells) was higher than that of the untypeable (n = 2, 0.271 copies/100 cells) and indeterminate samples (n = 1, 0.017 copies/ 100 cells), but there were no significant differences among them as the sample size were too small (Fig. 3). Most of the LIA-indeterminate samples were determined to be qPCR-negative, probably due to the very low proviral load of the carriers or to cross-nonspecific reactions with other antigens. Notably, samples with higher COI value detected by Roche showed higher PVLs but the there is no significant correlation between PVLs and cut-off ratios of Murex-ELISA and Roche-ECLIA (Additional file 1: Table S1).

## Discussion

The qPCR is one of the most important molecular confirmatory tools for HTLV-1/2 diagnosis. Serological confirmatory assays for HTLV-1/2 (WB and LIA) are expensive and tend to provide indeterminate and untyped results. In China, no molecular assays for HTLV-1/2 diagnosis are available. The purpose of the present study was to develop and validate a duplex qPCR HTLV-1 assay to simultaneously detect the presence of HTLV-1 and the RPPH1 reference gene, so as to solve the problems related to time constraints, cost, and data absent about PVLs in HTLV-1 infected patients in China.

The *LTR*, *pol*, gag, *tax* and *rex* genes are the regions used most frequently for HTLV-1 DNA quantitation. But deletions or mutations in the *rex* region have been reported in HAM/TSP cases and defection of 5’LTR frequently occurred in ATL cases [15–17]. Therefore, we screened specific primers and probes from a range of primer–probe sets targeting the *pol* and *tax* gene, which are relatively conservative in HTLV-1. In our study, a set of primer–probe with the best specificity for *pol* gene was finally used for our later work. It should be emphasized that we also screened specific primers and probes for internal reference RPPH1 gene, rather than using commercial kits described in previous studies [12, 18], which helped reduce detection costs and avoided having to independently detect the reference gene to count the number of cells. After optimizing the concentrations and annealing temperature of the selected primers and probes, we validated that neither of the two sets of primer-probes affects each other's fluorescence signal in a single tube.

Previous research identified HTLV-1 PVLs using successive dilutions of reference extracted DNA from cell lines with known HTLV-1 virus copy numbers [9]. This protocol was time-consuming, inconvenient and required certain cell-culturing facilities, making it challenging to use in a clinical setting. Synthetic oligonucleotides were utilized by Bandeira et al. to create a standard curve for measuring HTLV-1 PVL, and they underlined the fact that obtaining and maintaining such reference material was less expensive than collecting DNA from lineages of HTLV-1-infected cells [19]. However, plasmids are easier to build the standard curves for reproducible results than synthetic oligonucleotides and are more stable over time. In addition, two identical plasmids separately contain the HTLV-1 and internal control gene were often utilized to measure the PVLs. Such protocol necessitates accurate dilution of the standard curve of each reference plasmid, otherwise the results will be difficult to interpret due to the minimal discrepancies between the dilution format. In our study, a pcDNA3.1(-)-H1-*pol*-*tax*-RPPH1 that contained one copy of the RPPH1 gene and HTLV-1 target region was constructed and used to establish the standard curves, which was simple to prepare, highly reproducible and stable over time.

In fact, the duplex qPCR established in our study demonstrated a broad linear dynamic range, presented acceptable efficiency and was capable of detecting low-level proviral load (1 copy/reaction). To evaluate the detection limit of the method for the real sample, Huh102 cells were serially diluted with Jurkat cells and then were detected by our duplex qPCR for 20 times. Our method was sensitive enough to detect 2.5 copies per 10,000 cells. Although earlier research using mqPCRs revealed greater sensitivity for detecting and quantifying HTLV-1 than the current study, those earlier studies detected the reference gene independently to estimate the number of cells. For instance, Moens et al. developed a triplex qPCR for simultaneous detections of 4 copies of HTLV-1 per 10^5^ cells and 2.5 copies of HTLV-2 per 10^5^ cells, using 5 specific target primer-probes sets and RPPH1 as reference gene [12]. However, the reference gene to quantify cells number was detected separately by using the commercial kit and five different dyes are restricted to equipment and reagents.Lee et al. developed one duplex qPCR assay using primers that targeting the *tax* region shared by HTLV-1 and HTLV-2, and simultaneously detect the HLA-DQ alpha to normalize the amount of cellular DNA in another single qPCR tube; all of them based on SYBR Green dye. Although they displayed excellent sensitivity (1 copy per reaction), the duplex qPCR could not able to distinguish HTLV-1 from HTLV-2 and two tubes were required for each sample [20]. Another duplex qPCR developed by Estes and Sevall also used a singleplex assay to detect human β-globin for PVL quantification [21].As for the specificity, our duplex assay disclosed 100% specificity when applied in samples of patients infected with TP or HBV or HCV or HIV or HTLV-2 infected cell lines. Regarding the reproducibility, both intra- and inter-assays showed that the method developed in this study was surprisingly reproducible for the high, medium, and low plasmid concentrations, with CV of Ct values for HTLV-1 lower than 1.88 and 3.06%, respectively, and for RPPH1 lower than 1.95 and 2.90%, respectively. The accuracy was evaluated using independent extractions of standard Hut102 dilutions with accurate HTLV-1 proviral loads (quantitated by ddPCR). The analysis of six independent extractions within the same qPCR run showed samll variations for samples with high PVL. It is crucial to note that although though the PVL of the diluted Huh102 was as low as 0.026 copies/100 cells, the results detected by our method (0.0275 ± 0.0092 copies/100 cells) were very close to the theoretical values, reflecting a high accuracy.

In order to verify the feasibility of this method, LIA-positive and indeterminate samples with blood cells available were performed with WB and our duplex qPCR simultaneously. As shown in the results, 90.0% of LIA-positive, 33% of LIA-untypeable and 2% of LIA-indeterminate samples were confirmed as qPCR-positive, indicating that our qPCR method could successfully detect HTLV-1 in the majority of seropositive samples and was able to distinguish partial untypeable and indeterminate samples. The median PVL value of the qPCR-positive samples in our study was 1.621 copies/100 cells, which was either higher or lower or similar to previous reports [9, 22, 23]. In our study, HTLV-1-infected individuals with a broad range of PVLs, the values ranged from 0.001 to 11.397 copies per 100 PBMCs (Additional file 1: Table S1). This distribution was similar to the that observed in asymptomatic carriers (range: 0.018–12.434 copies/100 100 PBMCs) as well as in blood donors (about range: 0.001–10 copies/100 100 PBMCs approximated by their figures) [18, 22]. According to another study by Allison et al. [9], the PVL ranged from 0.0148 to 2.760 copies per 100 PBMCs in asymptomatic carriers. The conflicting results may be caused by the various geographic backgrounds and incomparable methodologies used in the various investigations. The median proviral load of LIA-positive samples measured by our method was approximately 100-fold higher than that of LIA-indeterminate and untypeable samples (1.780 copies/100 cells versus 0.186 copies/100 cells), which is in comparation to previous authors’ findings[18]. However, the number of samples with qPCR-positive but LIA-untypeable or indeterminate results in our study was too small, therefore, more samples need to be analyzed in the future to confirm this finding. It is interesting to note that one of the WB-indeterminate samples had a PVL of 11.4 copies per 100 cells, which was significantly higher than previously reported. However, Andrea et al. noted that one blood donor from Argentina had WB-indeterminate results while having a rather high PVL value (4.5copies/100 cells) [24]. This possibly due to the mutation in the HTLV-1 provirus, which reduces the expression of antigen and antibody. Such mutated sites resulting an antigenic alteration similar to what has been reported in cases of HBV infection [25].

In addition, 87% of LIA-positive, 17% of LIA-untypeable and none of the LIA-indeterminate samples were confirmed WB-positive in this investigation, demonstrating the duplex qPCR was more sensitive than WB. Actually, in our study, HTLV-1 provirus was found in 96.7% (27/28) of WB-positive blood donors, exceeding the detection rates of the qPCRs created by Moens et al. and Waters et al. for the seropositive blood donors (91% and 90%), and being close to the rate of a multiplex qPCR created by Gonçalves et al. (97.4%) [9, 10, 26]. However, the sensitivity was decreased to 74.6–77.1% when it employed in samples from HIV/HTLV-coinfected patients. The negative results may largely due to the use of highly active antiretroviral therapy (HAART), low proviral load, and punctual mutations in the genomic regions employed in PCR assays or defective provirus circulating in such patients [26, 27]. Unfortunately, our assay was not applied in such populations, but in the future, sensitivities of this assay in different populations should be analyzed. Furthermore, 33.3% (2/6) of WB-indeterminate samples were confirmed as qPCR-positive. This ratio is higher than those that have been previously recorded in other areas of the word, such as Brazil (9.2%), Iran (12.5%) and Argentina (14.7%) [28–30], but is lower than that in the United States (44%) [31], indicating the ratio may differ geographically. However, few data are available on the proportion of qPCR-positive samples among LIA-untypeable or indeterminate samples. LIA has a greater sensitivity and accuracy in comparison to WB for confirmation of HTLV-1/2 infection [7, 32]. But it usually yields more inconclusive results than WB, which may partial due to the very low proviral load of the carriers. Our findings also showed that the majority of the qPCR-negative samples had much lower Murex-ELISA and Roche-ECLIA cut-off values than the qPCR-positive samples, indicating that the inconclusive results may also due to cross-reactivity against other retroviruses or microbial agents, or to the seroconversion period. The real status of the blood donors need to be followed-up to ensure the blood safety.

In conclusion, the simplicity, applicable, reproducible and low-cost characteristics of the present assay for PVL quantification make it potentially an effective tool to instead of WB as the first-line test in confirming the ELISA reactive samples. It also offers a way for better compare of the PVLs obtained from different laboratories, and the results obtained support its use for monitoring infection and stratifying the risk for HTLV-1-associated disease.

## Supplementary Information

**Additional file 1**. Details of results by LIA, WB, qPCR, Murex-ELISA and Roche-ECLIA.

## Data Availability

Not applicable.

## Abbreviations

HTLV-1/2: Human T-cell lymphotropic virus type 1/2
ELISA: Enzyme-linked immunosorbent assay
ECLIA: Electrochemiluminescence immunoassay
WB: Western blot
LIA: Line immunoassay
PVL: Proviral load
RPPH1: RNase P
