# Supplementary material for: Development and validation of a duplex real-time PCR for the rapid detection and quantitation of HTLV-1
Source: Virol J. 2023 Jan 17;20:9. doi: 10.1186/s12985-023-01970-y (PMC9843979; doi:10.1186/s12985-023-01970-y)
Supplement: Supplementary file 1 — Additional file 1. Details of results by LIA, WB, qPCR, Murex-ELISA and Roche-ECLIA. [file 12985_2023_1970_MOESM1_ESM.docx]

Supplementary Materials for

**Development and Validation of a duplex real-time PCR for the rapid detection and quantitation of HTLV-1**

Huimin Ji ^12^, Le Chang ^12^, Ying Yan^12^, Lunan Wang^123 ＃^

1. National Center for Clinical Laboratories, Beijing Hospital, National Center of Gerontology; Institute of Geriatric Medicine, Chinese Academy of Medical Sciences, Beijing, P. R. China

2. Beijing Engineering Research Center of Laboratory Medicine, Beijing Hospital, Beijing, P. R. China

3. Graduate School, Peking Union Medical College, Chinese Academy of Medical Sciences, Beijing, P. R. China

^＃^Corresponding Author:

Lunan Wang, Email: [lunan99@163.com](mailto:lunan99@163.com)

Tel.: + 86 10 85133609

**Table S1. Details of results by LIA, WB, qPCR, Murex-ELISA and Roche-ECLIA**

| NO. | Sample No. |  | ELISA-Murex |  | CLIA-Roche |  | INNO-LIA |  | WB |  | qPCR | | |
| --- | --- | --- | --- | --- | --- | --- | --- | --- | --- | --- | --- | --- | --- |
|  |  |  | S/CO |  | COI |  | Results |  | Results |  | HTLV-1 Ct | RPPH1 Ct | PVL (copies/100cells) |
| 1 | 1312 |  | 11.06 |  | 450.2 |  | HTLV-1 |  | HTLV-1 |  | 29.46 | 23.98 | 3.333 |
| 2 | 1626 |  | 11.98 |  | 427.9 |  | HTLV-1 |  | HTLV-1 |  | 31.29 | 22.5 | 0.339 |
| 3 | 1648 |  | 12.25 |  | 365.2 |  | HTLV-1 |  | HTLV-1 |  | 30.84 | 24.05 | 1.345 |
| 4 | 1757 |  | 13.39 |  | 364.6 |  | HTLV-1 |  | HTLV-1 |  | 27.54 | 21.48 | 2.278 |
| 5 | 1782 |  | 12.61 |  | 70.62 |  | HTLV-1 |  | HTLV-1 |  | 27.31 | 18.16 | 0.273 |
| 6 | 1785 |  | 13.94 |  | 277.4 |  | HTLV-1 |  | HTLV-1 |  | 29.10 | 23.46 | 3.001 |
| 7 | 1918 |  | 12.26 |  | 317.3 |  | HTLV-1 |  | HTLV-1 |  | 23.67 | 18.79 | 5.288 |
| 8 | 1922 |  | 12.52 |  | 287.7 |  | HTLV-1 |  | HTLV-1 |  | 27.29 | 20.18 | 1.11 |
| 9 | 2250 |  | 12.32 |  | 329.1 |  | HTLV-1 |  | HTLV-1 |  | 27.94 | 20.1 | 0.67 |
| 10 | 2251 |  | 15.35 |  | 415.8 |  | HTLV-1 |  | HTLV-1 |  | 28.80 | 21.22 | 0.795 |
| 11 | 2271 |  | 12.72 |  | 303.6 |  | HTLV-1 |  | HTLV-1 |  | 33.36 | 23.09 | 0.12 |
| 12 | 2362 |  | 14.15 |  | 465 |  | HTLV-1 |  | HTLV-1 |  | 32.43 | 19.92 | 0.026 |
| 13 | 2368 |  | 13.31 |  | 214.7 |  | HTLV-1 |  | HTLV-1 |  | 31.50 | 20.44 | 0.071 |
| 14 | 2394 |  | 13.37 |  | 213.1 |  | HTLV-1 |  | HTLV-1 |  | 29.70 | 21.9 | 0.678 |
| 15 | 2406 |  | 13.12 |  | 136.5 |  | HTLV-1 |  | HTLV-1 |  | 29.37 | 22.44 | 1.23 |
| 16 | 2412 |  | 12.23 |  | 235.5 |  | HTLV-1 |  | HTLV-1 |  | 39.93 | 22.77 | 0.0012 |
| 17 | 2541 |  | 12.55 |  | 458.2 |  | HTLV-1 |  | HTLV-1 |  | 32.80 | 19.29 | 0.013 |
| 18 | 2548 |  | 12.48 |  | 235.4 |  | HTLV-1 |  | HTLV-1 |  | 30.93 | 18.75 | 0.033 |
| 19 | 2550 |  | 12.24 |  | 371.7 |  | HTLV-1 |  | HTLV-1 |  | 26.07 | 19.77 | 1.956 |
| 20 | 2606 |  | 9.80 |  | 398.4 |  | HTLV-1 |  | HTLV-1 |  | 29.07 | 21.7 | 0.913 |
| 21 | 2613 |  | 13.89 |  | 361.8 |  | HTLV-1 |  | HTLV-1 |  | 27.93 | 22.18 | 2.815 |
| 22 | 2649 |  | 11.62 |  | 125.7 |  | HTLV-1 |  | HTLV-1 |  | 36.05 | 22.19 | 0.01 |
| 23 | 2701 |  | 10.84 |  | 136.6 |  | HTLV-1 |  | HTLV-1 |  | 31.27 | 21.11 | 0.132 |
| 24 | 2718 |  | 11.57 |  | 124.2 |  | HTLV-1 |  | HTLV-1 |  | 29.22 | 21.45 | 0.695 |
| 25 | 2722 |  | 12.51 |  | 176.3 |  | HTLV-1 |  | HTLV-1 |  | 25.22 | 20.74 | 6.886 |
| 26 | 2752 |  | 20.38 |  | 150.5 |  | HTLV-1 |  | HTLV-1 |  | 28.76 | 22.17 | 1.562 |
| 27 | 2280 |  | 11.50 |  | 132.3 |  | HTLV-1 |  | HTLV-1 |  | ND | 21.04 | Neg |
| 28 | 1705 |  | 11.69 |  | 77.8 |  | HTLV-1 |  | IND |  | 27.82 | 24.11 | 11.397 |
| 29 | 2467 |  | 0.34 |  | 3.26 |  | HTLV-1 |  | IND |  | ND | 23.21 | Neg |
| 30 | 2719 |  | 11.37 |  | 2.27 |  | HTLV-1 |  | Neg |  | ND | 18.21 | Neg |
| 31 | 2415 |  | 13.03 |  | 214 |  | HTLV |  | HTLV-1 |  | 34.41 | 27.39 | 1.11 |
| 32 | 1739 |  | 12.38 |  | 178.20 |  | HTLV |  | IND |  | 37.96 | 23.97 | 0.009 |
| 33 | 2519 |  | 7.15 |  | 8.32 |  | IND |  | Neg |  | 36.54 | 23.46 | 0.017 |
| 34 | 1441 |  | 0.55 |  | 11.95 |  | HTLV |  | Neg |  | ND | 20.49 | Neg |
| 35 | 1747 |  | 2.02 |  | 42.31 |  | HTLV |  | Neg |  | ND | 22.02 | Neg |
| 36 | 1295 |  | 1.02 |  | 0.90 |  | HTLV |  | Neg |  | ND | 20.23 | Neg |
| 37 | 1461 |  | 0.38 |  | 0.40 |  | HTLV |  | Neg |  | ND | 20.63 | Neg |
| 38 | 2253 |  | 8.59 |  | 28.18 |  | IND |  | IND |  | ND | 22.60 | Neg |
| 39 | 2346 |  | 10.44 |  | 21.07 |  | IND |  | IND |  | ND | 21.04 | Neg |
| 40 | 2697 |  | 10.34 |  | 8.17 |  | IND |  | IND |  | ND | 21.55 | Neg |
| 41 | 1342 |  | 1.51 |  | 171.5 |  | IND |  | Neg |  | ND | 29.33 | Neg |
| 42 | 1357 |  | 0.19 |  | 197.1 |  | IND |  | Neg |  | ND | 20.79 | Neg |
| 43 | 1439 |  | 0.56 |  | 27.3 |  | IND |  | Neg |  | ND | 21.67 | Neg |
| 44 | 1454 |  | 0.13 |  | 8.05 |  | IND |  | Neg |  | ND | 23.06 | Neg |
| 45 | 1619 |  | 4.21 |  | 1.03 |  | IND |  | Neg |  | ND | 22.10 | Neg |
| 46 | 1621 |  | 2.66 |  | 1.37 |  | IND |  | Neg |  | ND | 24.08 | Neg |
| 47 | 1627 |  | 0.89 |  | 2.3 |  | IND |  | Neg |  | ND | 24.14 | Neg |
| 48 | 1654 |  | 0.35 |  | 2.19 |  | IND |  | Neg |  | ND | 26.41 | Neg |
| 49 | 1666 |  | 1.12 |  | 1.75 |  | IND |  | Neg |  | ND | 24.22 | Neg |
| 50 | 1673 |  | 1.84 |  | 0.415 |  | IND |  | Neg |  | ND | 20.37 | Neg |
| 51 | 1675 |  | 0.34 |  | 3.77 |  | IND |  | Neg |  | ND | 22.84 | Neg |
| 52 | 1693 |  | 8.72 |  | 3.62 |  | IND |  | Neg |  | ND | 25.13 | Neg |
| 53 | 1700 |  | 6.45 |  | 12.71 |  | IND |  | Neg |  | ND | 25.67 | Neg |
| 54 | 1733 |  | 1.95 |  | 0.485 |  | IND |  | Neg |  | ND | 22.93 | Neg |
| 55 | 2215 |  | 1.30 |  | 0.648 |  | IND |  | Neg |  | ND | 23.28 | Neg |
| 56 | 2338 |  | 1.39 |  | 0.289 |  | IND |  | Neg |  | ND | 21.48 | Neg |
| 57 | 2357 |  | 0.33 |  | 5.25 |  | IND |  | Neg |  | ND | 25.09 | Neg |
| 58 | 2364 |  | 0.44 |  | 4.77 |  | IND |  | Neg |  | ND | 25.62 | Neg |
| 59 | 2365 |  | 0.29 |  | 1.31 |  | IND |  | Neg |  | ND | 24.85 | Neg |
| 60 | 2386 |  | 9.28 |  | 1.43 |  | IND |  | Neg |  | ND | 23.60 | Neg |
| 61 | 2468 |  | 0.31 |  | 1.29 |  | IND |  | Neg |  | ND | 19.87 | Neg |
| 62 | 2474 |  | 0.70 |  | 32.28 |  | IND |  | Neg |  | ND | 21.20 | Neg |
| 63 | 2477 |  | 0.30 |  | 5.96 |  | IND |  | Neg |  | ND | 23.07 | Neg |
| 64 | 2478 |  | 7.75 |  | 0.362 |  | IND |  | Neg |  | ND | 19.88 | Neg |
| 65 | 2490 |  | 0.88 |  | 2.09 |  | IND |  | Neg |  | ND | 20.87 | Neg |
| 66 | 2492 |  | 0.08 |  | 3.04 |  | IND |  | Neg |  | ND | 20.74 | Neg |
| 67 | 2494 |  | 0.30 |  | 14.47 |  | IND |  | Neg |  | ND | 22.42 | Neg |
| 68 | 2653 |  | 1.52 |  | 0.979 |  | IND |  | Neg |  | ND | 20.72 | Neg |
| 69 | 2658 |  | 0.77 |  | 17.1 |  | IND |  | Neg |  | ND | 24.98 | Neg |
| 70 | 2659 |  | 0.37 |  | 4.72 |  | IND |  | Neg |  | ND | 20.14 | Neg |
| 71 | 2683 |  | 0.32 |  | 3.03 |  | IND |  | Neg |  | ND | 25.31 | Neg |
| 72 | 2688 |  | 0.25 |  | 14.61 |  | IND |  | Neg |  | ND | 20.50 | Neg |
| 73 | 2706 |  | 1.11 |  | 0.574 |  | IND |  | Neg |  | ND | 23.89 | Neg |
| 74 | 2714 |  | 2.92 |  | 0.512 |  | IND |  | Neg |  | ND | 23.49 | Neg |
| 75 | 2716 |  | 5.14 |  | 3.9 |  | IND |  | Neg |  | ND | 22.08 | Neg |
| 76 | 2729 |  | 4.87 |  | 0.786 |  | IND |  | Neg |  | ND | 19.08 | Neg |
| 77 | 2744 |  | 0.30 |  | 2.08 |  | IND |  | Neg |  | ND | 20.05 | Neg |
| 78 | 2745 |  | 0.42 |  | 2.39 |  | IND |  | Neg |  | ND | 18.61 | Neg |
| 79 | 2759 |  | 0.37 |  | 71.91 |  | IND |  | Neg |  | ND | 21.55 | Neg |
| 80 | 2781 |  | 0.59 |  | 13.93 |  | IND |  | Neg |  | ND | 21.19 | Neg |
| 81 | 2801 |  | 0.87 |  | 0.565 |  | IND |  | Neg |  | ND | 23.72 | Neg |
| 82 | 2826 |  | 9.92 |  | 47.55 |  | IND |  | Neg |  | ND | 25.90 | Neg |
| 83 | 2828 |  | 0.41 |  | 2.74 |  | IND |  | Neg |  | ND | 19.88 | Neg |
| 84 | 2830 |  | 0.89 |  | 25.6 |  | IND |  | Neg |  | ND | 20.51 | Neg |
| 85 | 2837 |  | 1.00 |  | 14.37 |  | IND |  | Neg |  | ND | 18.81 | Neg |

Note: WB, Western blot; LIA, line immunoassay; Ind, indeterminate, Pos, Positive; Neg, Negative;
